# DNA Repair Biosensor-Identified DNA Damage Activities of Endophyte Extracts from *Garcinia cowa*

**DOI:** 10.3390/biom10121680

**Published:** 2020-12-16

**Authors:** Tassanee Lerksuthirat, Rakkreat Wikiniyadhanee, Sermsiri Chitphuk, Wasana Stitchantrakul, Somponnat Sampattavanich, Siwanon Jirawatnotai, Juangjun Jumpathong, Donniphat Dejsuphong

**Affiliations:** 1 Research Center, Faculty of Medicine Ramathibodi Hospital, Mahidol University, Bangkok 10400, Thailand; tassanee.ler@mahidol.ac.th (T.L.); sermsiri.chi@mahidol.ac.th (S.C.); wasana.sti@mahidol.ac.th (W.S.); 2 Section for Translational Medicine, Faculty of Medicine Ramathibodi Hospital, Mahidol University, Bangkok 10400, Thailand; wiki.rakkreat@gmail.com; 3 Siriraj Center of Research for Excellence (SiCORE) for Systems Pharmacology, Department of Pharmacology, Faculty of Medicine Siriraj Hospital, Mahidol University, Bangkok 10700, Thailand; somponnat.sam@mahidol.ac.th (S.S.); siwanon.jir@mahidol.ac.th (S.J.); 4 Center of Excellent in Research for Agricultural Biotechnology and Department of Agricultural Science, Faculty of Agriculture, Natural Resources and Environment, Naresuan University, Phitsanulok 65000, Thailand

**Keywords:** *Garcinia cowa* Roxb. ex Choisy, endophyte, DNA damage and repair, cancer, biosensor

## Abstract

Recent developments in chemotherapy focus on target-specific mechanisms, which occur only in cancer cells and minimize the effects on normal cells. DNA damage and repair pathways are a promising target in the treatment of cancer. In order to identify novel compounds targeting DNA repair pathways, two key proteins, 53BP1 and RAD54L, were tagged with fluorescent proteins as indicators for two major double strand break (DSB) repair pathways: non-homologous end-joining (NHEJ) and homologous recombination (HR). The engineered biosensor cells exhibited the same DNA repair properties as the wild type. The biosensor cells were further used to investigate the DNA repair activities of natural biological compounds. An extract from *Phyllosticta* sp., the endophyte isolated from the medicinal plant *Garcinia cowa* Roxb. ex Choisy, was tested. The results showed that the crude extract induced DSB, as demonstrated by the increase in the DNA DSB marker γH2AX. The damaged DNA appeared to be repaired through NHEJ, as the 53BP1 focus formation in the treated fraction was higher than in the control group. In conclusion, DNA repair-based biosensors are useful for the preliminary screening of crude extracts and biological compounds for the identification of potential targeted therapeutic drugs.

## 1. Introduction

It is well known that conventional chemotherapy relies on inhibiting cell proliferation. Although this mode of action can target cancer cells, the side effects are frequently intolerable and treatment is not optimally effective in all patients [1]. Over the past decade, new therapies have been developed that use the molecular profiling of the patient’s tumor to tailor the treatment, so-called targeted therapy [2]. Targeting DNA double strand break (DSB) repair pathways has proved to be effective in cancer treatments [3,4]. Two major DNA DSB repair pathways are homologous recombination (HR) and non-homologous end-joining (NHEJ). Olaparib is the well-known example of targeted drugs to treat ovarian or breast cancer patients with *BRCA* germline mutations [5]. Olaparib has been approved by the U.S. FDA since 2014 and has been used as a single treatment or in combination with genotoxic drugs, such as cisplatin, to prolong the survival rate [6,7,8]. However, targeted drugs are expensive and the choice is often limited [9,10]. Additionally, current models for drug development are limited in terms of the identification and targeting of DNA repair activity.

Natural products have been the major source of anticancer drugs for more than 50 years [11]. They can be obtained from plants, microbes, and marine organisms. Recently, much attention has been devoted to investigating medicinal plants and their fungal endophytes for anticancer treatments [12,13,14]. Endophytes are microorganisms that inhabit the living tissue of a plant. Their relationship with the host plant can be symbiotic or slightly pathogenic [15,16]. Fungal endophytes have been reported to produce diverse types of secondary metabolites used in medicine, agriculture, and industry. Moreover, it has been stated that parts of the metabolites from medicinal plants might come from the endophyte’s metabolites [12,17,18]. For example, taxol and camptothecin (CPT) are well-established anticancer drugs that were initially isolated from the wood bark of *Taxus brevifolia* and *Camptotheca acuminata*, respectively [13,19]. Thereafter, it was found that isolated endophytic fungi from those trees could produce relevant plant metabolites [20,21,22,23].

*Garcinia cowa* Roxb. ex Choisy (Cha-muang in Thai) is a tropical tree found in South, East, and Southeast Asia [24,25]. Parts of the tree have been used for traditional medicine, as a food and for their ingredients [26,27,28,29]. Crude extracts and pure chemical constituents, i.e., cowanin, demonstrate different biological activities, such as antimalarial, antiviral, antifungal, antibacterial, antioxidant, anti-inflammatory, antiplatelet aggregation, and anticancer activities [30,31,32,33,34,35,36,37,38,39,40,41,42]. Although *Garcinia cowa* extracts have been extensively studied in cancer cell lines [40,41,43,44,45], the bioactivity against DSB repair pathways in host plants and endophytes has not been explored.

Because targeting DSB repair pathways is a promising target for cancer treatment, several researchers have developed the DSB biosensor to study the preference between HR and NHEJ. Mao et al. developed three fluorescent reporter assays to study the contribution of HR and NHEJ in each cell cycle phase of a human cell line [46,47]. Another study by Chien et al. generated a multiplexed bioluminescent repair reporter which can measure both HR and NHEJ activities in a single cell [48]. Despite these two biosensors being valuable to investigate the pathway choice in human cells, they might not be suitable for screening compounds due to the genome of cancer cell lines being mostly unstable over longer periods. Thus, a model with a more stable genetic background is of interest.

In this study, we created a biosensor based on DSB repair pathways from the DT40 cell line. DT40 has been well documented as a cellular model to study DNA damage and repair [49,50,51,52]. DNA repair-deficient DT40 cell lines are widely used in high-throughput screening to evaluate the genotoxicity of substances or find compounds affecting DNA repair [53,54,55,56]. The lack of p53 in DT40 causes the cell to pass through the G1-S checkpoint, thereby allowing DNA damage accumulated through the cell cycle and repair mechanism to be investigated. In our experiment, fluorescent protein (FP) was tagged with 53BP1 C-terminus, a protein representing NHEJ, and RAD54L C-terminus, a protein representing HR. The biosensor was validated for the repair activity and was used to test the crude extracts from *Garcinia cowa* and its endophyte. Our results demonstrate an effective tool for screening natural extracts for DNA-damaging properties.

## 2. Materials and Methods

### 2.1. Cell Lines and Culture Conditions

Wild-type DT40 and DNA repair-deficient cell lines, *RAD54L*^−/−^ [57], *53BP1*^−/−^ [58], and *LIG4*^−/−^ [59], were kindly provided by Dr. Shunichi Takeda (Department of Radiation Genetics, Graduate School of Medicine, Kyoto University, Kyoto, Japan). Both wild-type DT40 and knockout cells were maintained at 40 °C under 5% CO_2_ in RPMI-1640 medium (Gibco, Thermo Fisher Scientific, MA, USA) supplemented with 10% fetal bovine serum (Merck, Darmstadt, Germany), 1% chicken serum (Gibco), 2 mM L-glutamine (Gibco), 100 µM β-mercaptoethanol (Gibco), and 100 unit/mL penicillin/streptomycin (Gibco) [60].

### 2.2. Construction of the Fluorescent Biosensor Clones

**Figure 1:**
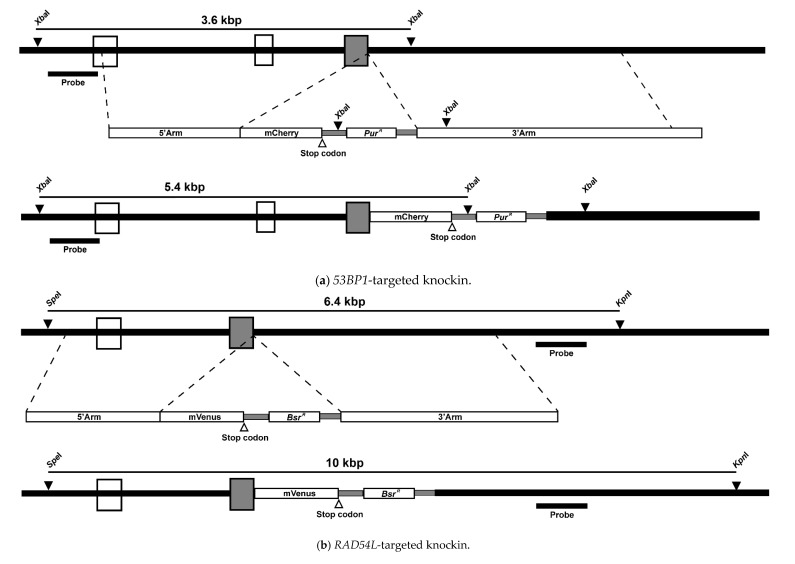

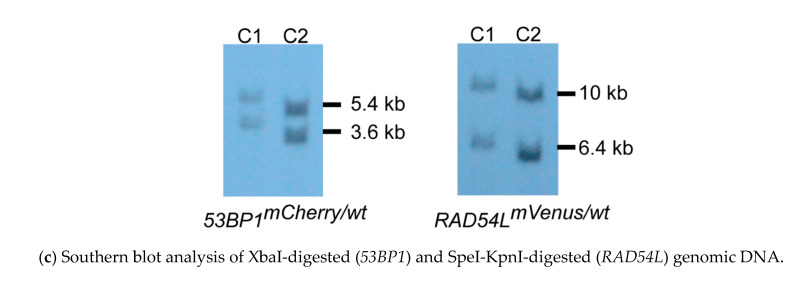
Construction of fluorescent biosensor clones. (**a**) mCherry was inserted at the C-terminus of *53BP1*. The non-targeted and targeted knockin of *53BP1* when the genomic DNA was cut with XbaI were 3.6 and 5.4 kbp. (**b**) For *RAD54L*, the non-targeted and targeted knockin when the genomic DNA was cut with SpeI and KpnI were 6.4 and 10 kbp. Codon regions are represented by boxes, and the gray-colored box is the last codon in each gene. The black triangle is the restriction site, and an open triangle denotes the stop codon. (**c**) Southern blotting of XbaI-digested (53BP1) and SpeI-KpnI-digested (RAD54L) genomic DNA. Definitions: mCherry, monomeric Cherry; mVenus, monomeric Venus; kbp, kilobase pair; *53BP1*, tumor protein p53 binding protein 1; *RAD54L*, DNA repair protein RAD54-like; WT, wild type.

To construct the targeted knockin, the 3′- and 5′-arms of *53BP1* (Gene ID: 415589) were amplified from the genomic DNA. Then, the fluorescence tag mCherry was amplified from the pPB_FoxM1b D-box-116 del Mut No.6 plasmid (kindly provided by Dr. Sampattavanich) and designed to have a stop codon at its C-terminus. Both amplicons (the 53BP1 and mCherry) were later assembled with the pre-restriction cut (SpeI and KpnI) pBluescript II SK(+) using NEBuilder HiFi DNA Assembly Cloning Kit (New England Biolabs, Ipswich, MA, USA). Then, the assembled construct was inserted with a selectable marker cassette: puromycin acetyltransferase (Pur, Figure 1a). The targeted knockin for *RAD54L*-mVenus (Gene ID: 424611) was also constructed using a similar approach (Figure 1b) [61]. Before transfection, the constructs were linearized with NotI. The transfection protocol was followed as previously described [62]. Furthermore, 0.5 and 25 µg/mL puromycin dihydrochloride (Toku-E, Zwijnaarde, Belgium) and blasticidin S HCl (Toku-E) were used to select the transfected clones. Targeted knockin clones were screened using Southern blotting with the PCR probe, designed within the XbaI to detect positive clones in the transfected *53BP1,* and SpeI and KpnI to detect clones in the transfected *RAD54L* (Figure 1). The primers used in this study are summarized in Table 1.

### 2.3. Proliferation Determination

To investigate the proliferation rate, 1 × 10^4^ cells were seeded in a 24-well plate in 1 mL culture medium. Viable cells were counted under the microscope by observing the Trypan blue exclusion at a 24-hr interval using a hemocytometer.

### 2.4. Focus Formation by Immunofluorescence

Here, 5–7 × 10^5^ cell/mL were continuously exposed to 100 nM CPT (Sigma, St. Louis, MO, USA) for 1 hr. The cells were spun on the slide, then fixed with 4% paraformaldehyde in PBS, after which the cells were permeabilized by 0.1% Triton X-100 in PBS, and non-specific binding was blocked by the Odyssey^®^ blocking buffer. Rabbit monoclonal antibody against γH2AX, Ser139 (CST, Danvers, MA, USA), rabbit polyclonal IgG antibody against RAD51 (H-92) (SantaCruz, Dallas, TX, USA), mouse monoclonal antibody against RAD51 (SantaCruz), and rabbit polyclonal anti-53BP1 antibody (Novus Biologicals, Centennial, CO, USA) were used to detect γH2AX and 53BP1 focus formation, respectively. Alexa Fluor 647 donkey anti-rabbit IgG (Thermo Fisher Scientific) and Alexa Fluor 647 donkey anti-mouse IgG (Thermo Fisher Scientific) were used as the secondary antibody. The nuclei were counter-stained using Hoechst 33342 (Thermo Fisher Scientific). Images were acquired using the Operetta High-Content Analysis System (PerkinElmer, Waltham, MA, USA). At least 300 cells were counted by eye in images opened in ImageJ 1.52p [63]. Positive foci formation of γH2AX, 53BP1, and RAD54L cells were determined by more than four foci per cell.

### 2.5. Plant Extraction

*Garcinia cowa* Roxb. ex Choisy (Cha-Muang) leaves were harvested from Pitsanulok Province, Thailand in October 2018. The leaves were identified by visually comparing the specimen with the herbarium database (http://www.phargarden.com, Faculty of Pharmacy, Ubon Ratchathani University, Thailand). The extraction of crude metabolites from the leaves was adapted from Tayana et al. [40]. Briefly, 10 g of leaves was washed and blended into small pieces. Then, 30 mL of either ethanol or ethyl acetate was added and incubated for 7 days in the dark to obtain the crude metabolites. The leaves were filtered and the plant-crude extract was dried using a rotary evaporator at 45 °C. The dried-crude extract was later solubilized in dimethyl sulfoxide (DMSO) before use. *Garcinia cowa* Roxb. ex Choisy is written as *Garcinia cowa* in short.

### 2.6. Isolation of Endophytic Fungi from Garcinia cowa

The isolation of endophytic fungi from the *Garcinia cowa* leaf was adapted from Wiyakrutta et al., and Potshangbam et al. [64,65]. Briefly, the leaves were cleaned with running faucet water and sterilized by sequentially soaking in 4–6% sodium hypochlorite solution for 1 min, 70% ethanol for 1 min, and sterile distilled water three times. The leaves were cut into small pieces, which were placed on half-strength potato dextrose agar (PDA) and yeast malt agar (YMA) with 100 µg/mL *chloramphenicol* (Calbiochem, San Diego, CA, USA). The plates were incubated at 30 °C for 14 days until hyphal growths were observed. To identify the fungal species, genomic DNA was sequenced using the internal transcribed spacer (ITS) 1–4 primers (Macrogen, Seoul, South Korea), and subsequently analyzed by NCBI blast (https://blast.ncbi.nlm.nih.gov). The sequence was submitted to GenBank under no. MK530204.1.

### 2.7. Fermentation Condition and Metabolite Extraction

To prepare the seed inoculum, 10 pieces of 1 cm × 1 cm actively growing hyphal were transferred to potato dextrose broth and incubated at 30 °C for 48 h with shaking. Then, 10% of seed inoculum was transferred to modified Wickerhams Antibiotic Test Medium and incubated at 30 °C for 5 days [66]. The fungal mass was filtered to obtain the cultured medium (secretory metabolite). Both secretory and mycelia crude extract metabolites were obtained using ethyl acetate and dried using a rotary evaporator at 45 °C. The dried crude extract was later solubilized in DMSO before use.

### 2.8. Determination of Viability with DNA Repair-Deficient Cell Line Panels

The viability assay was adapted from Yomamoto et al. and Wiyakrutta et al. [56,64]. DT40 and DNA repair-deficient cell lines were seeded into 96-well plates at a density of 40,000 cells/mL in the final volume of 100 µL. Thereafter, 50 µL of various concentrations of crude extracts was added to each well and the cells were incubated at 40 °C with 5% CO_2_ for 24–48 h. The final DMSO concentration was set to not exceed 0.3% (*w/w*). The cell viability was measured using CellTiter-Glo^®^ (Promega, Madison, WI, USA) and the signal was read out using the Infinite 200Pro microplate reader (Tecan, Männedorf, Switzerland).

### 2.9. Statistical Analysis

All graphs show the mean ± standard deviation (SD), and statistically significant differences were analyzed using Student’s *t*-test with GraphPad Prism 7 (GraphPad Software, Inc., San Diego, CA, USA).

## 3. Results

### 3.1. Fluorescent Biosensor Construction and Proliferative Properties of Biosensor Clones

**Figure 2:**
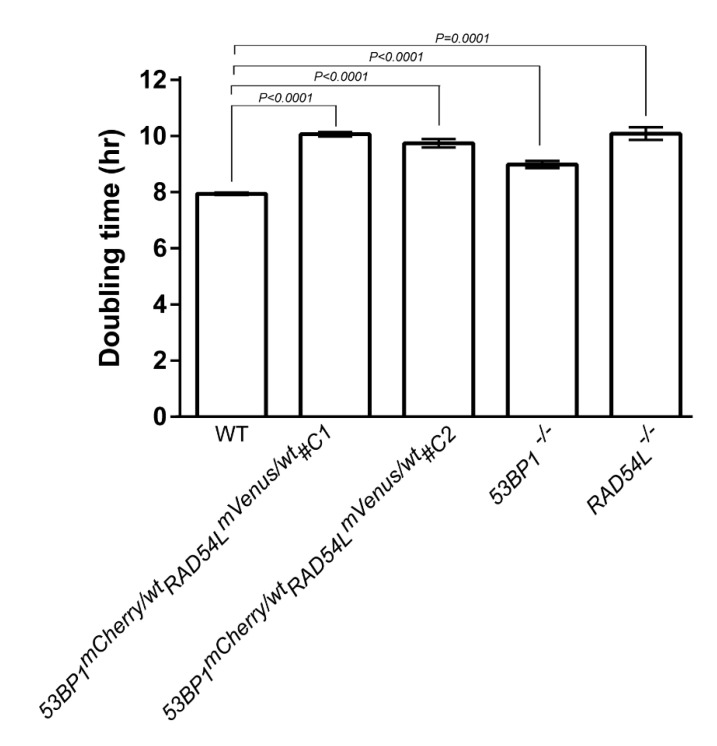
Cell proliferation of biosensor clones was slightly lower than wild-type DT40. Two clones of targeted knockin were generated and they demonstrated the same phenotype. Data are shown as the mean ± standard deviation of three biologically independent experiments. Definitions: 53BP1-mCherry-RAD54L-mVenus#C1, tumor protein p53 binding protein-monomeric Cherry-RAD54-like-monomeric Venus knockin clone 1; 53BP1-mCherry-RAD54L-mVenus#C2, tumor protein p53 binding protein-monomeric Cherry-RAD54-like-monomeric Venus knockin clone 2; *53BP1*, tumor protein p53 binding protein 1; *RAD54L*, DNA repair protein RAD54-like.

We designed plasmids to replace the stop codon of DNA repair genes with the fusion fluorescent proteins mCherry and mVenus in the *53BP1* and *RAD54L* genes, respectively. Heterologous targeted knockin of both genes at the endogenous 3′-end was confirmed by Southern blotting (Figure 1). The accomplishment of *53BP1-mCherry-RAD54L-mVenus* targeted knockin was indicated by the presence of two bands at 3.6 kb (WT) and 5.4 kb (mCherry) for 53BP1 and at 6.4 kb (WT) and 10 kb (mVenus) for RAD54L. Two clones of the *53BP1-mCherry-RAD54L-mVenus* DNA repair biosensor cell were selected and the proliferative rates of both clones were significantly slower than those of the wild type (Figure 2). The doubling times of the wild type, *53BP1-mCherry-RAD54L-mVenus#C1, 53BP1-mCherry-RAD54L-mVenus#C2, 53BP1*^−/−^, and *RAD54L*^−/−^ were 7.95 ± 0.02, 10.07 ± 0.08, 9.75 ± 0.15, 10.09 ± 0.23, and 8.99 ± 0.13 h, respectively.

### 3.2. DNA Repair Phenotypic Validation of Biosensor Clones

**Figure 3:**
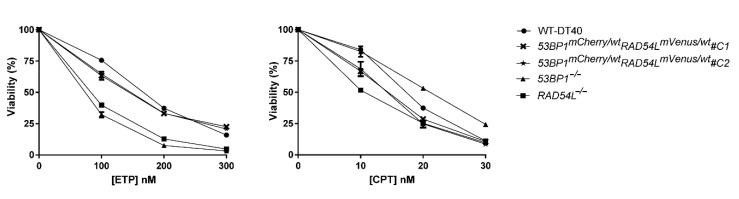
The viability of biosensor clones following exposure to DNA-damaging agents demonstrated the same phenotype as wild-type DT40. The experiments were reproduced with biologically independent replicates. Data are shown as the mean ± standard deviation from one experiment. The error bars are shorter than the height of the symbol in some points. Definitions: ETP, etoposide; CPT, camptothecin.

To validate the phenotype of the biosensor cells, we performed a viability assay of the biosensor clones following exposure to the DNA-damaging agents CPT and etoposide (ETP) (Figure 3). Both chemotherapeutic drugs generated DNA DSB that required HR and NHEJ repair pathways, respectively. *53BP1*^−/−^ and *RAD54L*^−/−^ cells were used as the positive control in this experiment. Both biosensor clones showed a similar viability to the wild type in the ETP-treated group. However, the biosensor clones demonstrated a lower viability at 10 nM CPT, though a higher viability than the *RAD54L*^−/−^ clones. When the concentration of CPT increased to 20 nM, the viability of the biosensors clone was at a similar level to that of the *RAD54L*^−/−^ clones. These results demonstrated that heterozygous wild-type/fluorescent biosensor alleles function similarly in DNA repair and could represent a biosensor for DNA repair activities. Since biosensor clones 1 and 2 showed the same phenotype on both the cellular proliferation and viability assay, clone 1 was selected for further experiments and designated *53BP1-mCherry-RAD54L-mVenus*.

### 3.3. Expression of Fluorescent mCherry 53BP1 and mVenus-RAD54L Proteins

To confirm the expression and localization of biosensors, we tested cells with CPT and ETP to detect HR and NHEJ activities, respectively. CPT, a widely used chemotherapeutic agent, is a topoisomerase I (topo I) inhibitor and specifically generates DSBs during the S-phase, which requires error-free HR [67]. Notably, observing the focus formation of RAD51 and RAD54L reflects the HR activity since RAD51 and RAD54L are key effectors in HR.

ETP is a topo II inhibitor and affects the religation step of the topo II cleavage complex, resulting in DSBs [68]. 53BP1 protein is the DNA damage response protein and promotes NHEJ repair [69]. Consequently, visualizing 53BP1 focus formation could represent the activity of NHEJ.

**Figure 4:**
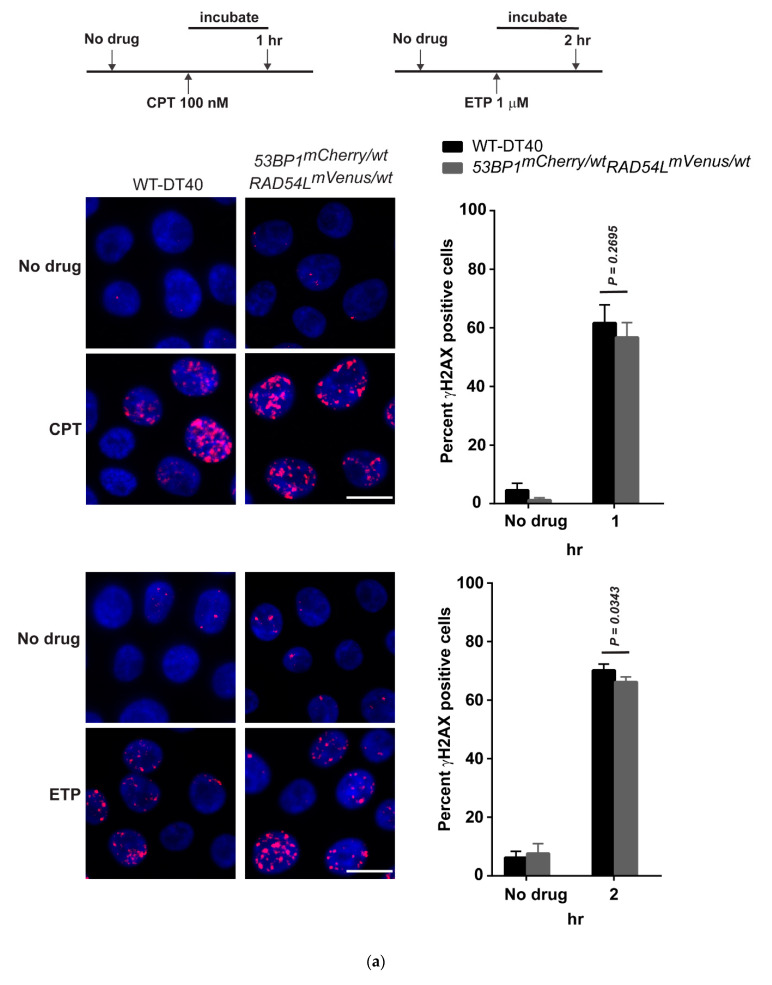

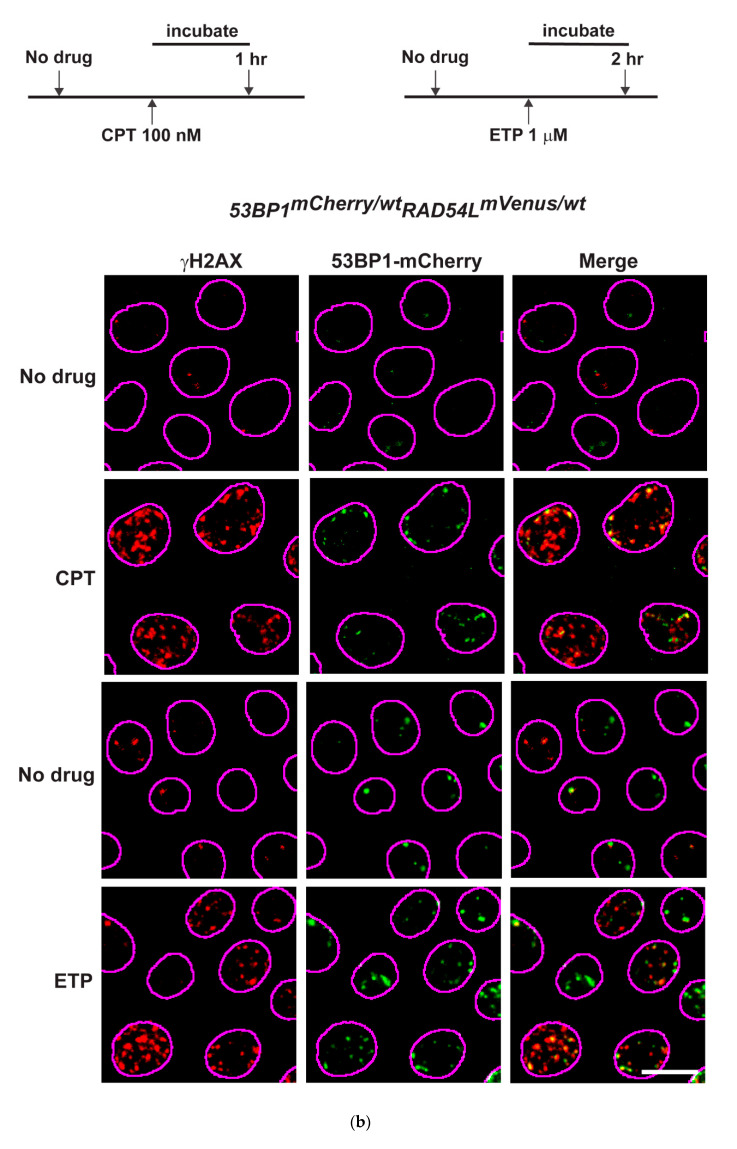

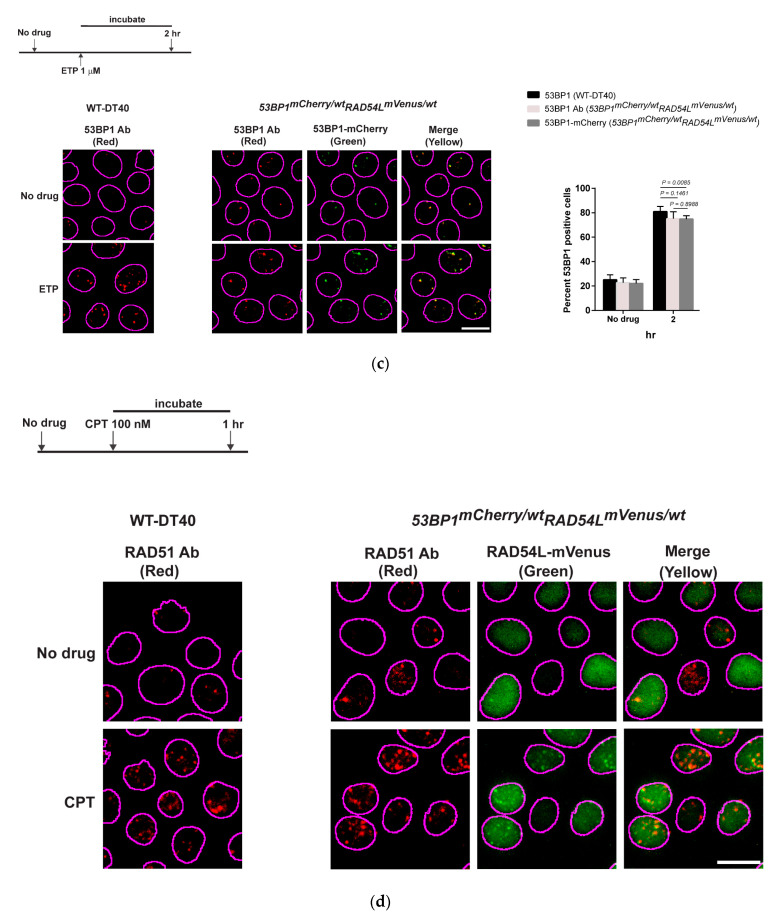
Foci formation of biosensor clones revealed the same phenotype as that of wild-type DT40. (**a**) γH2AX (red dot) focus formation of wild-type and biosensor cells when treated with either CPT or ETP. (**b**) Colocalization (yellow) of γH2AX (red) and 53BP1-mCherry (green) in wild-type and biosensor cells when treated with either CPT or ETP. (**c**) Colocalization (yellow) of 53BP1 antibody (red) and 53BP1-mCherry (green) in wild-type and biosensor cells when treated with ETP. (**d**) Colocalization (yellow) of the RAD51 antibody (red) and 53BP1-mCherry (green) in wild-type and biosensor cells when treated with CPT. The bar graphs are shown as the mean ± standard deviation. Definitions: 53BP1-mCherry-RAD54L-mVenus, tumor protein p53 binding protein-monomeric Cherry-RAD54-like-monomeric Venus knockin cells; WT, wild type, ETP, etoposide; CPT, camptothecin; Ab, antibody. Scale bar represents 10 μM.

After incubation with 100 nM CPT for 1 h or 1 µM ETP for 2 h to introduce DNA damage, the *53BP1-mCherry-RAD54L-mVenus* clone had a similar percentage of γH2AX positive cells as the wild type, with *p =* 0.2695 (CPT) and *p* = 0.0343 (ETP) (Figure 4a). The similarity of foci formation of phosphorylated histone H2AX (γH2AX) indicated normal DSB repair activation in the biosensor cells [70]. Next, we tested whether the mCherry-53BP1 biosensor overlapped with γH2AX. Following 2 h of treatment with 1 µM ETP, mCherry-53BP1 appeared to colocalize with γH2AX (Figure 4b) as well as endogenous 53BP1 (Figure 4c). The percentages of 53BP1 positive cells were similar in the wild-type and biosensor cells (*p* = 0.1461) when staining with the 53BP1 antibody, and the number of overlapping foci in the biosensor cells in endogenous 53BP1 (antibody staining) and 53BP1-mcherry was > 90% in both the no-drug and the ETP-treated group (Figure 4c).

The *53BP1-mCherry-RAD54L-mVenus* cells were treated with 100 nM CPT for 1 h to verify RAD54L-mVenus as a biosensor of HR. The result showed that RAD51 foci overlapped with RAD54L, indicating sites of HR, and the number of overlapping RAD51 and RAD54L foci was > 90% (Figure 4d). These showed that our HR and NHEJ biosensors performed similarly in terms of localization and quantitation to endogenous proteins after DNA damage.

### 3.4. Biological Properties of Crude Extracts from Garcinia cowa

**Figure 5:**
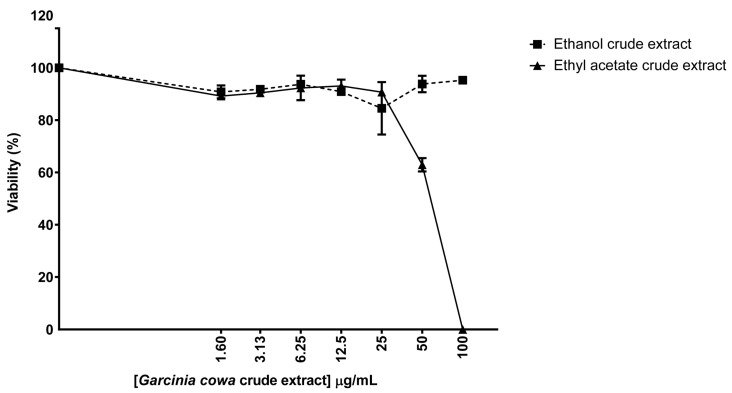
Ethyl acetate crude extracts from *Garcinia cowa* Roxb. ex Choisy affected the biosensor cell viability. The viability assay of the biosensor cells was measured following exposure with either *Garcinia cowa* Roxb. ex Choisy ethyl acetate or ethanol crude extract for 48 h. The experiment was repeated with biologically independent replicates. Each point is shown as the mean ± standard deviation from one experiment.

The viability assay was used to determine the effect of two crude extracts, ethanol and ethyl acetate, on biosensor cell survival. The cells were continuously exposed to *Garcinia cowa* crude extracts for 48 h. The results showed that the crude extracts from ethanol did not affect the biological activities of the biosensor cells even when the cells were treated with up to 100 μg/mL (Figure 5). The viability of the ethanol crude extract group ranged from 84% to 100%. However, viability started to decrease when the cells were exposed to the ethyl acetate crude extracts. Specifically, the viability was reduced to 63% at 50 μg/mL and 0.03% at 100 μg/mL. It is probable that the ethyl acetate fraction contained chemical constituents that were toxic to the cells. Therefore, in further experiments, ethyl acetate was chosen to extract the crude metabolites from the isolated endophytes.

### 3.5. Identification of Fungal Endophyte from Garcinia cowa

**Figure 6:**
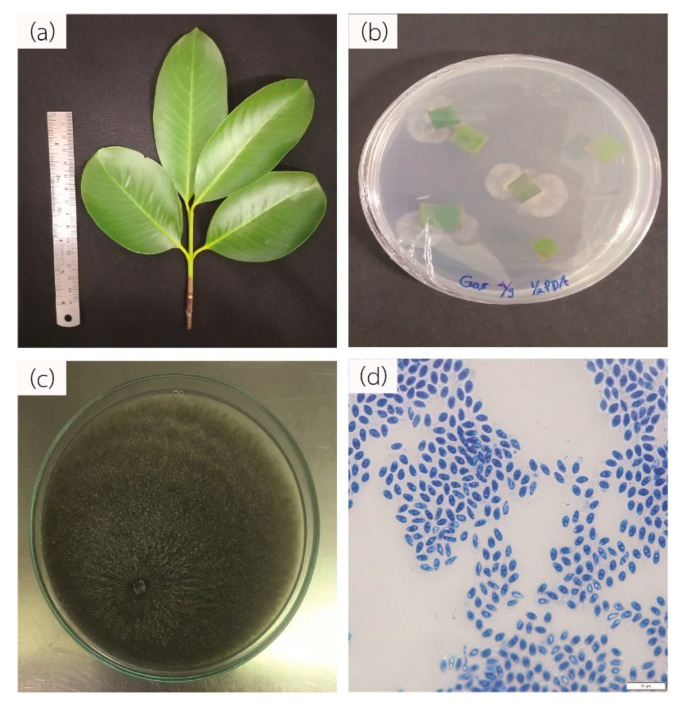
Overall endophytic fungi isolation. (**a**) *Garcinia cowa* Roxb. ex Choisy leaves. (**b**) Morphology of *Phyllosticta* sp. on half strength potato dextrose agar (PDA) and (**c**) yeast malt agar (YMA). (**d**) Morphology of *Phyllosticta* sp. spore. The scale bar represents 20 µm.

Forty-one microbial isolates were obtained from the healthy leaves of *Garcinia cowa* (Figure 6a). The gross morphology and microscopic appearance were all identical. They appeared as a green fluffy growth on the PDA plate with shuttle-shaped spores (Figure 6b,c). We selected one isolation for further experiments. Genomic DNA was extracted and the fungus was identified using ITS1-4 as the standard primers. The ITS1-4 sequence was 95% identical with the *Phyllosticta ampelicida* or *Guignardia bidwellii* isolate G17 ITS sequence (HM049170.1). Therefore, we denoted endophyte as *Phyllosticta* sp. YGE41.

### 3.6. Biological Properties of Crude Extracts from Phyllosticta sp. YGE41

**Figure 7:**
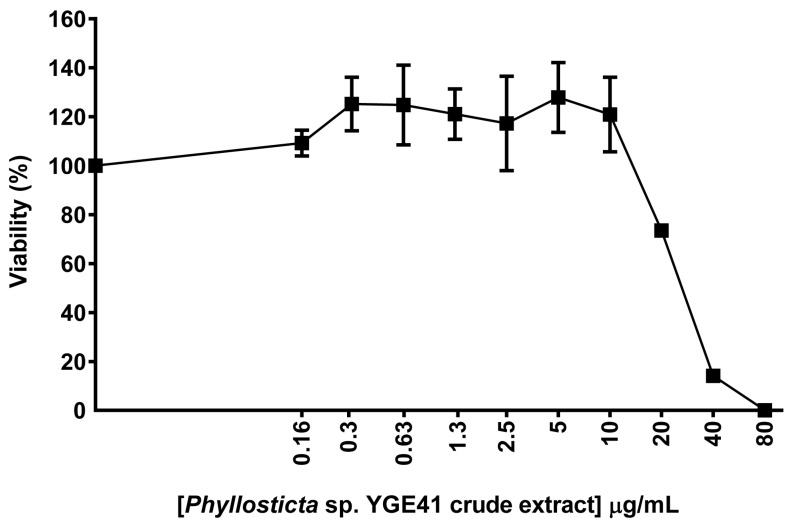
*Phyllosticta* sp. YGE41 crude extracts affected the viability of biosensor cells. Thebiosensor cells were treated continuously with ethyl acetate crude metabolites from *Phyllosticta* sp. YGE41 for 48 h before viability determination. The viability assay was repeated with biologically independent replicates. Each point is shown as the mean ± standard deviation from one experiment.

The viability assay was used to determine the effect of ethyl acetate crude extracts from *Phyllosticta* sp. YGE41. The result demonstrated that the ethyl acetate extracts from both *Phyllosticta* sp. YGE41 and *Garcinia cowa* affected biosensor cell viability (Figure 5 and Figure 7). The viability of the biosensor treated with *Phyllosticta* sp. YGE4 ethyl acetate crude extract was reduced to 74%, 14%, and 0.02% when the cells were treated at 20, 40, and 80 μg/mL, respectively (Figure 7). We selected 10 μg/mL to further investigate DNA damage and repair activity at the molecular level.

### 3.7. Garcinia cowa Extract-Induced DNA DSBs Damage through 53BP1

**Figure 8:**
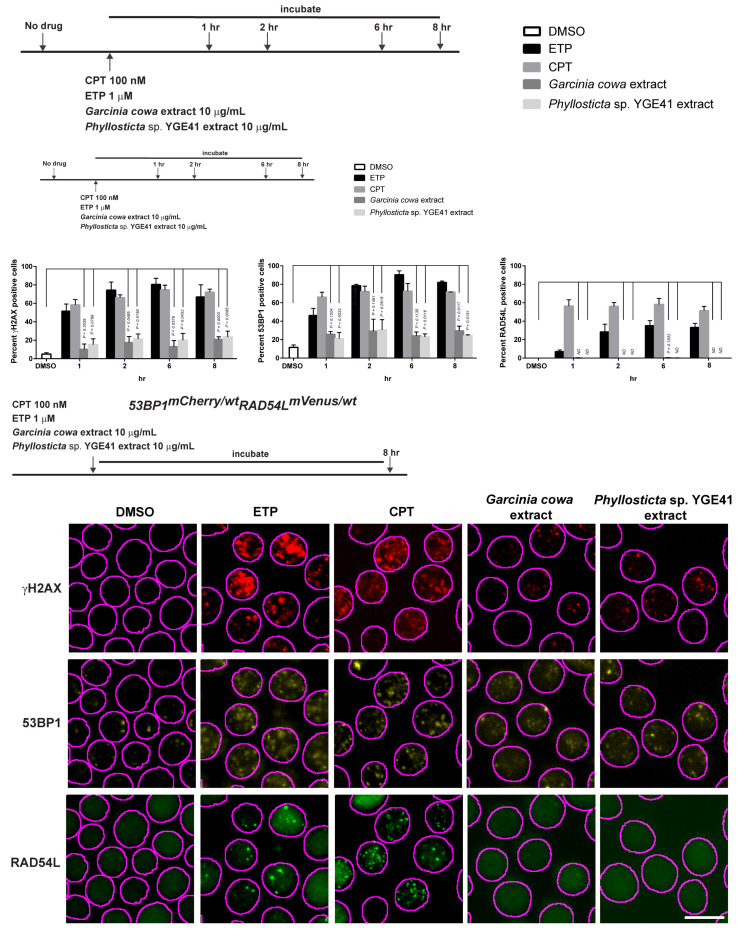
*Garcinia cowa* and *Phyllosticta* sp. YGE41 crude extracts caused mild DSB and activated NHEJ. The biosensor cells were continuously treated with the crude extracts for 8 h and were collected at the indicated time. The bar graphs are shown as the mean ± standard deviation. The foci formation images are represented at 8 h. Definitions: DMSO, dimethyl sulfoxide; ETP, etoposide; CPT, camptothecin; ND, Student’s *t*-test cannot be determined.

The biosensor cells were continuously treated with either *Garcinia cowa* or *Phyllosticta* sp. YGE41 for 8 h. During the treatment, the cells were collected to observe the focus formation according to each time point represented in Figure 8. In this experiment, CPT and ETP were included in the assay as positive controls, and DMSO was used as a negative control. The result showed that *Garcinia cowa* and *Phyllosticta* sp. YGE41 extracts induced moderate DSBs. The values of γH2AX-positive cells at 8 h were 21% and 24%, respectively, whereas the value for the DMSO control was 5%. The values of 53BP1 positive cells at 8 h were 28% for *Garcinia cowa* and 27% for *Phyllosticta* sp. YGE41, and the value for the DMSO control was 11%. There was no RAD54L focus formation observed from either the *Garcinia cowa* and *Phyllosticta* sp. YGE41 extracts. The focus formation of 53BP1 and RAD54L suggested that the damage caused by the extracts activated NHEJ rather than HR.

### 3.8. Viability of Crude Extract with NHEJ-Deficient Cells

**Figure 9:**
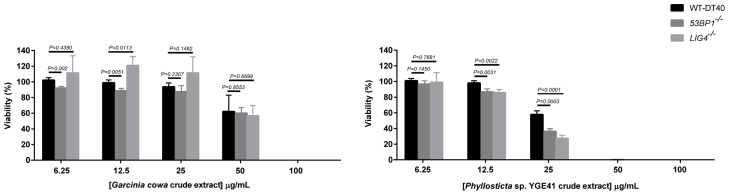
*Garcinia cowa* and *Phyllosticta* sp. YGE41 crude extracts differentially affected NHEJ-deficient cells. The cells were treated continuously with ethyl acetate crude metabolites from *Garcinia cowa* Roxb. Ex Choisy and *Phyllosticta* sp. YGE41 to determine cell viability. The experiment was repeated with biologically independent replicates. Data are shown as the mean ± standard deviation. Student’s *t*-test was performed at 6.25, 12.5, 25, and 50 μg/mL of *Garcinia cowa* and *Phyllosticta* sp. YGE41. Definitions: DMSO, dimethyl sulfoxide; *53BP1*^−/−^, *53BP1* knockout; *RAD54L*^−/−^, *RAD54L* knockout.

We further explored whether the crude extracts affected the downstream level of NHEJ, since 53BP1 is the initial player in the NHEJ pathway. LIG4 is the major protein in the last step of NHEJ by which it ligates the broken ends of DSBs [71]. The viability profiles of both *53BP1*^−/−^ and *LIG4*^−/−^ treated with *Garcinia cowa* extract were similar to the wild type. Thus, 53BP1 and LIG4 did not seem to play a role in *Garcinia cowa* extract-induced DNA damage repair (Figure 9). In contrast to *Phyllosticta* sp. YGE41 treatment, both *53BP1*^−/−^ (36.52%) and *LIG4*^−/−^ (27.31%) had a lower viability than the wild type (57.75%) at 25 µg/mL (Figure 9). Consequently, *Phyllosticta* sp. YGE41-induced DNA damage appeared to contain metabolites participating in NHEJ. The potency of the crude extract from *Phyllosticta* sp. YGE41 was higher than that of *Garcinia cowa* Roxb. ex Choisy, as was evidenced by the fact that the viability was almost obliterated for *Phyllosticta* sp. YGE41 extract at 50 µg/mL, whereas for *Garcinia cowa* Roxb. ex Choisy extract, it was significantly diminished at 100 µg/mL.

## 4. Discussion

In this study, we constructed a biosensor based on the DSB repair pathways to investigate the bioactivity, with particular focus on DNA damage and repair, of crude extracts from the medicinal plant *Garcinia cowa* and its endophyte *Phyllosticta* sp. YGE41. The DSB repair reporter was previously developed as a biosensor to detect HR or NHEJ in human cell lines. Mao et al. employed a rare-cutting endonuclease sequence, I-SceI, to generate the plasmid cassettes to separately determine HR and NHEJ [46,47]. Chien et al. improved the DSB repair reporter, by developing a plasmid cassette to simultaneously detect HR and NHEJ within the same cells [48]. These reporters are very useful tools to study the pathway choice of DSB in particular cell lines. However, the genetic stability of the human cancer cell model is not stable over longer periods and so might not be suitable for screening compound libraries. DT40, a chicken B lymphoma cell line, is known for its stable karyotype and is used in DNA repair studies [49,72]. Thus, it is a model of choice in the development of cell-based biosensors to determine DSB repair activity.

Two representative proteins were selected because studies have reported the use of the targeted knockin of FP. Pedersen et al. and Oestergaard et al. tagged FP to the C-terminus of 53BP1 to study the functions of TopBP1 and RNF8, RNF168, and HER2 during DNA damage, respectively [73,74]. Eppink et al. and Agarwal et al. attached green FP to the C-terminus to study the function of RAD54 in HR [75,76]. Thus, it was conceivable that the fusion of two fluorescent proteins, mCherry and mVenus, would not disrupt the repair function. Our results showed that biosensor cells grew at a slower rate compared to the wild type. However, in the viability assay with ETP and CPT, DNA-damaging agents, the viability profiles of both clones were comparable with the wild type. Both clones showed the same phenotype in the cell growth and viability assays, so we selected clone #1 for further experiments.

We determined the subnuclear focus formation of γH2AX, 53BP1, RAD54L, and RAD51 to observe DNA damage and repair at the molecular level [77]. Our results showed that the biosensor cell had the same phenotype as the wild type. In terms of quantity, the biosensor clone had the same focus formation of γH2AX (Figure 4a) and 53BP1 (Figure 4c). Moreover, 53BP1 colocalized with γH2AX after DNA-induced damage by either CPT or ETP (Figure 4b). This suggested that DSB response and NHEJ repair in biosensor cells were the same as in the wild type. We used the RAD51 antibody to confirm the HR activity of RAD54L in biosensor cells since no antibody against chicken RAD54L was commercially available. RAD54L is a helicase and interacts with RAD51, facilitating the strand invasion [78]. Our colocalization results for RAD51 and RAD54L confirm that biosensor cells retained the HR activity (Figure 4d).

Natural products such as plants and endophytic fungi are the source of various anticancer drugs [79]. *Garcinia cowa* was of interest because reports have shown that its chemical constituents affected cancer growth [39,80]. Moreover, we were interested in its endophyte due to the possibility of producing the same metabolites as the host plant and novel metabolites of pharmaceutical importance [12,81]. Different solvents yielded different metabolic profiles [82]. Thus, biosensor cells were used to preliminarily determine the bioactive metabolic profiles from *Garcinia cowa* leaves in ethyl acetate and ethanol. Our results demonstrate that the ethyl acetate-derived crude metabolites possessed dose-dependent biological activity toward biosensor cells (Figure 5). Endophytes were isolated from *Garcinia cowa* leaves, which induced 41 isolated endophytes. Our results show that 41 isolates had the same hyphae and spore morphology. Thus, one isolate was used as a representative and was later identified to belong to *Phyllosticta* sp. The ITS1-4 sequence identity was between *Phyllosticta ampelicida/Guignardia bidwellii* and the isolate was 95%. 

Although many studies have considered the viability effects of secondary metabolites from *Garcinia cowa* on cancer cell lines [40,41,80,83,84], none has investigated the biological activity from the endophytes isolated from *Garcinia cowa* leaves. Few reports reveal the biological activity of *Phyllosticta ampelicida/Guignardia bidwellii* metabolites from other host plants [85]. Xia et al. mechanistically investigated the pure active compounds from *Garcinia cowa* leaves on apoptosis, autophagy, and cell cycle arrest [80]. *Phyllosticta ampelicida*/*Guignardia bidwellii* is a fungal plant pathogen that produces phytotoxic metabolites, for example, phenguignardic acid, guignardic acid, alaguignardic acid, and guignardianones A, E, and F. Guignardic and phenguignardic acids have no toxicity against HeLa S3 or Jurkat cells. No mechanistic study on DNA damage and repair has been considered, probably because of the unavailability of DNA repair biosensors. Our results demonstrate that both extracts from *Garcinia cowa* and *Phyllosticta* sp. YGE41 caused mild DSB, which was validated by γH2AX increment (Figure 8). 53BP1 was also higher in the crude metabolite-treated group, showing that the damaged DNA activated NHEJ repair.

Typically, a panel of DNA repair-deficient cell lines is used to mechanistically elucidate the DNA-repairing process following exposure to genotoxic agents [68]. When examining the crude extracts with NHEJ repair-deficient cell lines, both extracts showed a difference in viability profiles (Figure 9), showing that each extract might have a distinct metabolic profile with similar mild DNA damage properties. Damage induced by the *Garcinia cowa* extract did not seem to be repaired by 53BP1 and LIG4, as the viability profile in each corresponding knockout was similar to the wild type (Figure 9). At 25 μg/mL of *Phyllosticta* sp. YGE41 extract, both *53BP1*^−/−^ and *LIG4*^−/−^ showed a different change in cell viability compared with the wild type. Unlike the *Garcinia cowa* extract, 53BP1 and LIG4 seemed to participate in repairing the damage induced by *Phyllosticta* sp. YGE41 extract. Additionally, the killing potency of *Phyllosticta* sp. YGE41 extract appeared to be greater than that of the *Garcinia cowa* extract. Further experiments on the purification of a positive fraction would provide more insight into the pure bioactive compounds affecting DNA damage and repair.

## 5. Conclusions

We used DT40 cell lines to develop a biosensor based on the DSB repair pathways. The repair activity of the biosensor was analyzed, and it was used to screen the crude metabolites from *Garcinia cowa* Roxb. ex Choisy leaves and its endophyte, *Phyllosticta* sp. Crude extracts from the ethyl acetate fraction induced DNA damage and the repair process. Thus, our biosensor could be used as an effective tool for bioassay-guided purification.

## Figures and Tables

**Table 1:** List of primers used in this study.

Name	5′ to 3′ Direction	Description
pBlue-5′Arm53BP	CACCGCGGTGGCGGCCGCTCTAGAAAATACTTGCTGTGCCTTG	5′-Arm 53BP1
mTur-BglII-5′Arm53BP	CCTTGCTCACCATACGAGGGACATAGTCATG	5′-Arm 53BP1
5′Arm-mCher53BP	CTATGTCCCTCGTATGGTGAGCAAGGGCGAG	FP-53BP1
3′Arm-mCher53BP	GCAGGACAGCAGCAGATCTTTACTTGTACAGCTCGTCC	FP-53BP1
mTur-BglII-3′Arm53BP	CAAGTAAAGATCTGCTGCTGTCCTGCTGTCC	3′-Arm 53BP1
pBlue-3′Arm53BP	ACTAAAGGGAACAAAAGCTGGGTACGTTGTTTTGGGGATAGGTAGAAG	3′-Arm 53BP1
pBlu-5′ArmRad54	CGGTGGCGGCCGCTCTAGAAATCCACTCCTGTGGTCAC	5′-Arm Rad54
mVenus-BamHI-5′ArmRad54	TGCTCACCATGGGAATCCCTCGCTGCTC	5′-Arm Rad54
5′Arm-mVenusRad54	AGGGATTCCCATGGTGAGCAAGGGCGAG	FP-Rad54
3′Arm-mVenusRad54	AGTTAACCTGGGATCCTTATTTGTACAATTCGTCC	FP-Rad54
mVenus-BamHI-3′ArmRad54	ATAAGGATCCCAGGTTAACTGTCCCCTG	3′-Arm Rad54
3Arm-Rad54PrimerR1	AGGGAACAAAAGCTGGGTACCATTTCTCTCCTCTTAGGG	3′-Arm Rad54L
Rad54-Probe	TGCAGTCCTTTCAGTGGCTA	Southern blot probe
Rad54-Probe	CAGCCTTTAACTGGGTCAGC	Southern blot probe
53BP-Probe	ACATTGGCTGGTTGGATCTC	Southern blot probe
53BP-Probe	GTGTTCGACAATGCTGATCC	Southern blot probe
ITS1 5′	TCCGTAGGTGAACCTGCGG	Internal transcribed spacer
ITS4 5′	TCCTCCGCTTATTGATATGC	Internal transcribed spacer
